# Development of ethical competences in mental health and psychiatry: simulation with nursing students

**DOI:** 10.1192/j.eurpsy.2023.1860

**Published:** 2023-07-19

**Authors:** R. Lopes, I. Moreira, M. Neves, C. Brás, R. Rodrigues

**Affiliations:** Escola Superior de Enfermagem de Coimbra, Coimbra, Portugal

## Abstract

**Introduction:**

Simulation as a pedagogical strategy contributes to improving the acquisition, consolidation and retention of knowledge and is very attractive for students.

In simulation learning, students come into contact with real clinical practices, allowing them to develop personal, psychosocial, ethical and clinical skills, facilitating learning for decision-making.

The creation of different and complex simulation scenarios within the scope of Mental Health and Psychiatry (MHP) allows the empowerment of nursing students, through the anticipation and prevention of errors and the creation of training opportunities, which culminate in the development of critical thinking and reflective on the ethical dimension of caring for people experiencing mental illness.

**Objectives:**

To analyze the simulation as a strategy to develop ethical competences in MHP; To reflect on respect for autonomy, capacity for self-determination and dignity of the person; To reflect on care practices that promote respect and dignity for people experiencing mental illness.

**Methods:**

After the careful design of the situation simulation scenario in MHP, the steps are as follows:

Prebriefing - transmit generic information about the scenario to the participants/students; request the participation of some students to assume the role of actors in the context they will encounter and prepare to start the case; explain to observers what will happen and the goals of the scenario.

- Scenario development.
- Debriefing - ask observers to analyse and reflect on positive aspects of performance; lead participants to analyse and reflect on their actions; investigate the basis of gaps/errors.
- Reflection - facilitating students’ structured thinking (reflection-in-action and reflection-on-action); review learning.
- Assessment - focus group interview; observation and/or filming.

**Results:**

The evaluation revealed that the use of a simulation scenario allows the connection between the theoretical contents of ethics (principles, dignity of the person, human rights, informed consent, …) with what they saw and experienced in the scenario; facilitates understanding of concepts, helps to internalize knowledge and retain information; favors reflection, development of critical thinking through discussion and argumentation; makes it easier to understand the relationship between the subject taught and reality; and the discussion of the situation helped to structure the thought.

The diversity of scenarios is interesting and useful, it allows understanding the different role of nurses in the hospital context and in the context of primary care.

**Conclusions:**

It is concluded that the use of a simulation scenario in MHP is of great interest and usefulness for the development of ethical competences, allowing reflection on care practices that promote respect and dignity of the person with experience of mental illness.

**Disclosure of Interest:**

None Declared

